# Pan-Cancer Analysis Identified Homologous Recombination Factor With OB-Fold (HROB) as a Potential Biomarker for Various Tumor Types

**DOI:** 10.3389/fgene.2022.904060

**Published:** 2022-07-12

**Authors:** Xianming Liu, Cunchuan Wang

**Affiliations:** ^1^ Department of Gastrointestinal Surgery, Shenzhen People’s Hospital, The Second Clinical Medical College of Jinan University, The First Affiliated Hospital of Southern University of Science and Technology, Shenzhen, China; ^2^ Department of Gastrointestinal Surgery, Guangzhou Overseas Chinese Hospital, The First Affiliated Hospital of Jinan University, Guangzhou, China

**Keywords:** pan-cancer, HROB, stemness, prognosis, biomarker

## Abstract

**Background:** By recruiting the MCM8–MCM9 helicase to DNA damage site, the Homologous Recombination Factor With OB-Fold (HROB) is involved in the repair of inter-strand crosslink and homologous recombination. Previous studies have shown that HROB may play an oncogenic role by promoting cell proliferation and chemoresistance in several tumor types. However, the potential diagnostic and prognostic values of HROB have not been systemically explored in pan-cancer.

**Methods:** We analyzed the expression pattern of HROB among tumor tissues and normal tissues in several public databases, including Human Protein Atlas and the Cancer Genome Atlas (TCGA) and investigated the association between the HROB expression and pathological stage and patient prognosis. We also analyzed the association between HROB expression and cancer stemness and immune infiltration of cancer-associated fibroblasts (CAFs) and CD8^+^ T cells in pan-cancer. Finally, we explored the potential biological function of HROB through pathway enrichment analysis.

**Results:** In most tumor types, HROB is overexpressed in tumor tissues compared with non-tumor tissues. High HROB expression was correlated with poor prognosis and advanced pathological stages. HROB expression was robustly correlated with cancer stemness. Moreover, significant correlations between CAFs, CD8^+^ T-cell infiltration, and HROB expression were observed in several tumor types. Pathway enrichment analysis revealed that cell cycle and mitotic-regulated pathways were strongly enriched in HROB co-expressed genes.

**Conclusion:** HROB may be a potential diagnostic and prognostic biomarker in pan-cancer, which may play a role in tumorigenesis and disease progression by affecting the cancer stemness of tumor tissues and immune cell infiltration.

## Introduction

According to the cancer morbidity estimated by GLOBOCAN 2020, approximately 19.3 million new cancer cases have occurred in 2020 around the world. Meanwhile, there were approximate 10.0 million cancer deaths worldwide in 2020, which demonstrated that high fatality rates are still shared by most tumor types (Sung et al., 2021). However, our understanding of tumorigenesis is still limited. Identification of potential oncogenes might help us to develop diagnostic biomarkers and treatment targets for cancer. In the last decade, large sample cancer datasets such as The Cancer Genome Atlas (TCGA) have provided the basis for characterizing novel oncogenes for different cancers (ICGC/TCGA Pan-Cancer Analysis of Whole Genomes Consortium, 2020; Lv et al., 2021; Ye et al., 2021; Cheng et al., 2021).

Homologous Recombination Factor With OB-Fold (HROB), also known as C17ORF53 or MCM8IP, was a novel gene reported by several studies related to inter-strand crosslink repair and homologous recombination (Hustedt et al., 2019; Huang et al., 2020; Wang et al., 2020). Molecular mechanism studies revealed that HROB promotes DNA synthesis by recruiting the MCM8–MCM9 helicase to DNA damage sites (Huang et al., 2020). A recent study also showed that HROB depletion could slow down the progression of DNA replication fork and cell proliferation (Wang et al., 2020). In addition, Huang et al. found that HROB was required for chemoresistance to cisplatin and other cancer therapeutic agents (Huang et al., 2020). Nicole et al. found that the HROB–MCM8–MCM9 pathway could control the homologous recombination (Hustedt et al., 2019). Moreover, genome-wide screens have identified HROB as a potential regulator for temozolomide and ATR inhibitor resistance (MacLeod et al., 2019; Wang et al., 2019). These evidences suggested that HROB may play an oncogenic role by promoting cell proliferation and chemoresistance in various tumor types. However, the predictive and prevalence value of HROB in tumors has not been systemically explored in pan-cancer.

In this study, we systematically investigate the predictive and prevalence value of HROB in pan-cancer. We also investigated the relationship between HROB expression and cancer stemness, immune cell infiltration of tumor tissues. Finally, we explored the HROB potential molecular function through constructing a network of HROB-related genes.

## Materials and Methods

### Gene Expression Analysis of *HROB*

The clinicopathological and transcriptome data of The Cancer Genome Atlas (TCGA) datasets were acquired from the UCSC Xena (http://xena.ucsc.edu).

Oncomine database (http://www.oncmine.org) was used to validate differential HROB expression between tumor tissues and non-tumor tissues in pan-cancer with the following thresholds: 1) *p* < 0.05 and 2) fold change >1.5; the HROB cell-type mRNA expression plot was generated by human protein atlas (HPA) database (https://www.proteinatlas.org/).

GTEx transcriptome data were acquired from the GTEx portal v8 (https://www.gtexportal.org/).

“Stage plot” module of GEPIA 2.0 (http://gepia2.cancer-pku.cn/) was used to generate pathological stage plots for TCGA tumor types.

### Survival Analysis

The patients were divided into high expression group and low expression group by HROB median expression. R package “survival” was used to perform the statistical analysis of survival data. R package “survminer” was used to visualize the results of survival analysis by generating the Kaplan–Meier curves (K–M). The prognostic significance of HROB expression regarding the overall survival (OS), disease-specific survival (DSS), and progression-free interval (PFI) was assessed by the cox regression model (Yu et al., 2021).

### Immune Cell Infiltration Analysis

“Immune” module of Tumor Immune Estimation Resource version 2 (TIMER2) (http://timer.cistrome.org/) was used to investigate the correlation between HROB expression and cancer-associated fibroblasts (CAFs), CD8^+^ T-cell infiltration by the Extended Poly-dimensional Immunome Characterization (EPIC) algorithm.

### HROB-Related Gene Enrichment Analysis

The main parameters of the STRING tool (https://string-db.org/) were used to generate the HROB co-expression network. For the network-type parameter, we chose the full network. For the meaning of the network edge parameter, we chose the confidence. For the active interaction source parameter, we chose co-expression. For the minimum required interaction score parameter, we chose the low confidence (0.150). For the max number of interactor parameter, we chose 50.

The “Most Similar Genes” module of GEPIA2 was used to obtain 100 *HROB*-co-expressed genes with the most similar expression pattern to *HROB* in the TCGA datasets and GTEx (Tang et al., 2019). These 100 genes were then analyzed with the R package “clusterProfiler” to conduct Gene Ontology (GO) and Kyoto Encyclopedia of Genes and Genomes (KEGG) pathway enrichment analysis.

## Result

### Analysis of HROB Gene Expression

**FIGURE 1:**
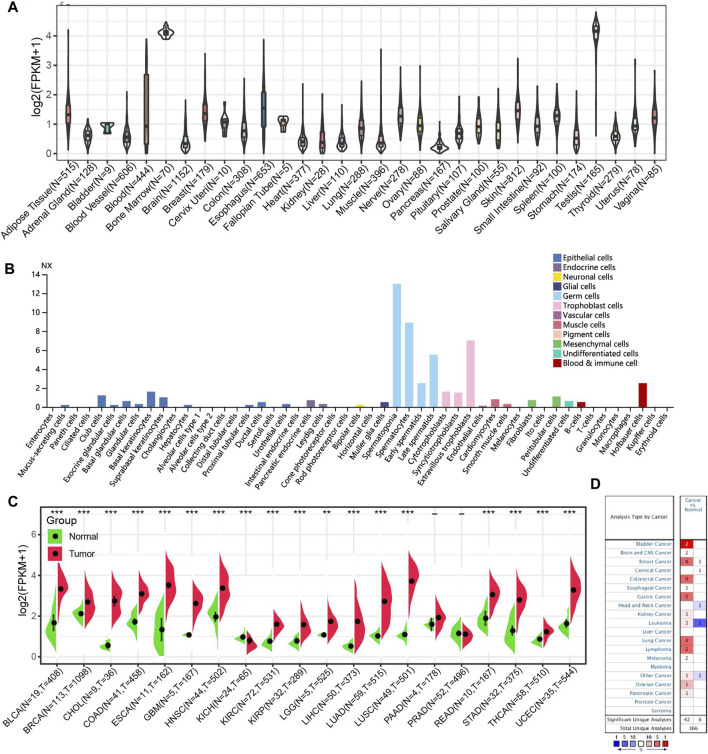
*HROB* expression level in TCGA tumors and normal tissues. **(A)** *HROB* tissue expression based on GTEx datasets. **(B)** HROB expression of various cell types. **(C)** The differential expression of HROB between tumor tissues and non-tumor tissues in TCGA datasets. *p < 0.05; **p < 0.01; and ***p < 0.001. **(D)** Oncomine pooling analysis of HROB expression in various tumor types with the following thresholds: i. *p* < 0.05; ii. fold change >1.5.

We first explored the HROB expression in normal tissues by analyzing GTEx transcriptome data and observed a higher HROB expression in testicular tissues and bone marrow tissues (Figure 1A). In addition, we also found that HROB enriched in germ cells of testicular origin by analyzing the cell-type RNA-seq (Figure 1B). In general, a weak tissue-specificity of HROB expression was observed in the testicular tissue. We then investigated the expression pattern between tumor tissues and non-tumor tissues from the TCGA datasets. We found that HROB exhibited significant higher expression in tumor tissues by comparing with the corresponding non-tumor tissues, including bladder urothelial carcinoma (BLCA), breast invasive carcinoma (BRCA), cholangiocarcinoma (CHOL), colon adenocarcinoma (COAD), esophageal carcinoma (ESCA), glioblastoma multiforme (GBM), head and neck squamous cell carcinoma (HNSC), kidney renal clear cell carcinoma (KIRC), kidney renal papillary cell carcinoma (KIRP), brain lower grade glioma (LGG), liver hepatocellular carcinoma (LIHC), lung adenocarcinoma (LUAD), lung squamous cell carcinoma (LUSC), rectum adenocarcinoma (READ), stomach adenocarcinoma (STAD), thyroid carcinoma (THCA), and uterine corpus endometrial carcinoma (UCEC) (Figure 1C). Meanwhile, we found that the HROB expression significantly decreased in kidney chromophobe (KICH) tumor tissues by comparing with the corresponding non-tumor tissues (Figure 1C).

To validate HROB differential expression between tumor and non-tumor tissues, we used Oncomine database to investigate the HROB expression pattern in other datasets. The pooling results of these datasets confirmed that HROB is overexpressed in various tumor types, though counterexamples have also been observed in several datasets (Figure 1D).

**FIGURE 2:**
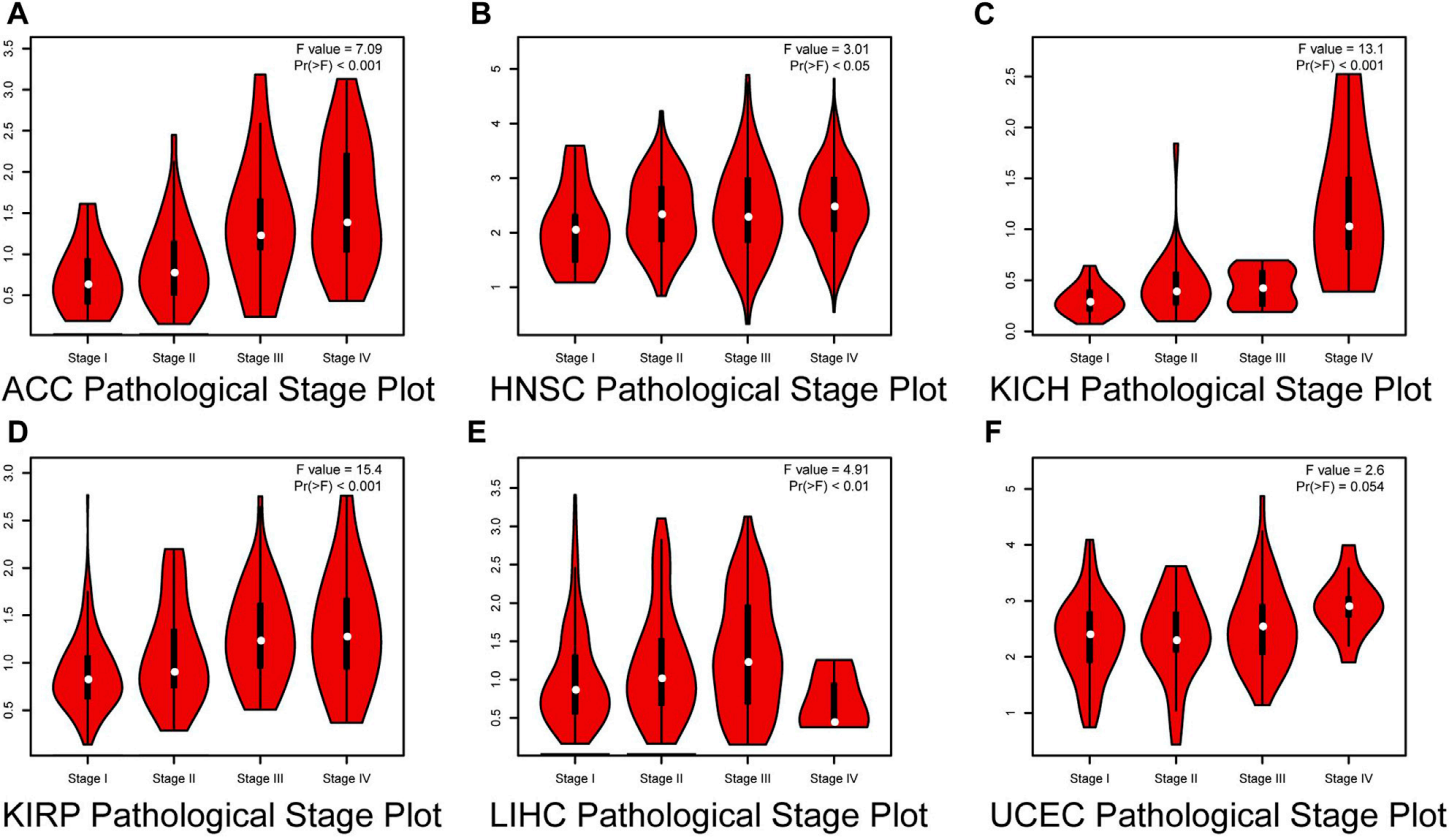
Correlation between *HROB* expression and pathological stages of **(A)** ACC, **(B)** HNSC, **(C)** KICH, **(D)** KIRP, **(E)** LIHC, and **(F)** UCEC from TCGA datasets.

We then investigated the correlation between HROB expression and pathological stage of different tumor types by GEPIA2. Our results showed that a high HROB expression was significantly correlated with the advanced stage of adrenocortical carcinoma (ACC), HNSC, KICH, LIHC, KIRP, and UCEC (Figure 2).

### High HROB Expression May Indicate Poor Prognosis for Cancer Patients

**FIGURE 3:**
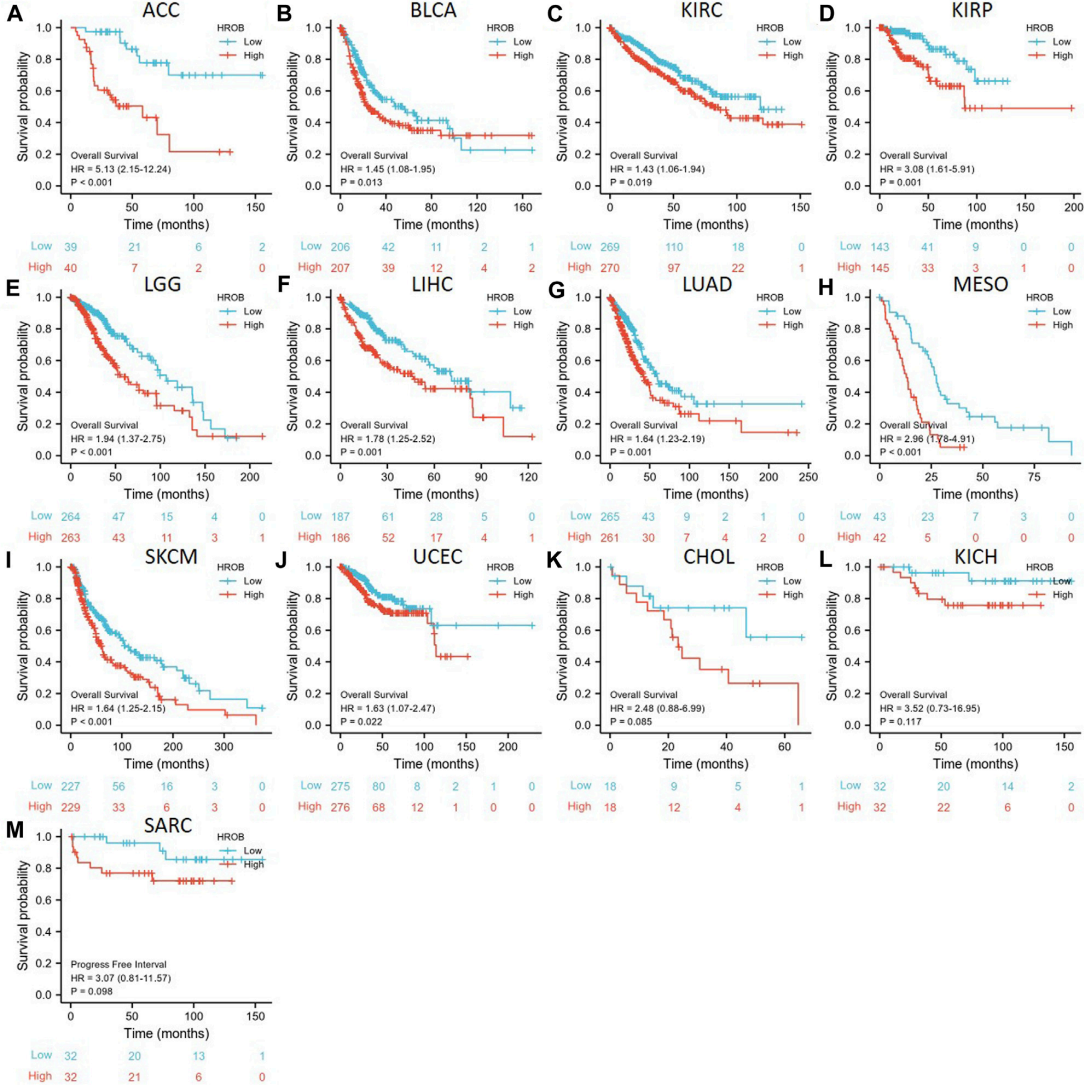
Correlation between *HROB* expression and overall survival in TCGA patients with various tumor types **(A–M)**.

By performing survival analysis in different tumor types, we investigated the potential prognostic value of HROB. First, we explored the correlation between HROB expression and OS in TCGA tumor patients. Our results revealed significant correlation between shorter OS and higher HROB expression in ACC (*p* < 0.001), BLCA (*p* = 0.013), KIRC (*p* = 0.019), KIRP (*p* = 0.001), LGG (*p* < 0.001), LIHC (*p* = 0.001), LUAD (*p* = 0.001), mesothelioma (MESO) (*p* < 0.001), skin cutaneous melanoma (SKCM) (*p* < 0.001), and UCEC (*p* = 0.022) patients (Figures 3A–J). In addition, similar correlations were also observed in CHOL (*p* = 0.075), KICH (0.117), and sarcoma (SARC) (0.087) patients (Figures 3K–M). Raw data for the OS analyses in Figure 3 can be found in the Supplementary Table S1. In addition, a summary table for all survival analyses can be found in Supplementary Table S2.

**FIGURE 4:**
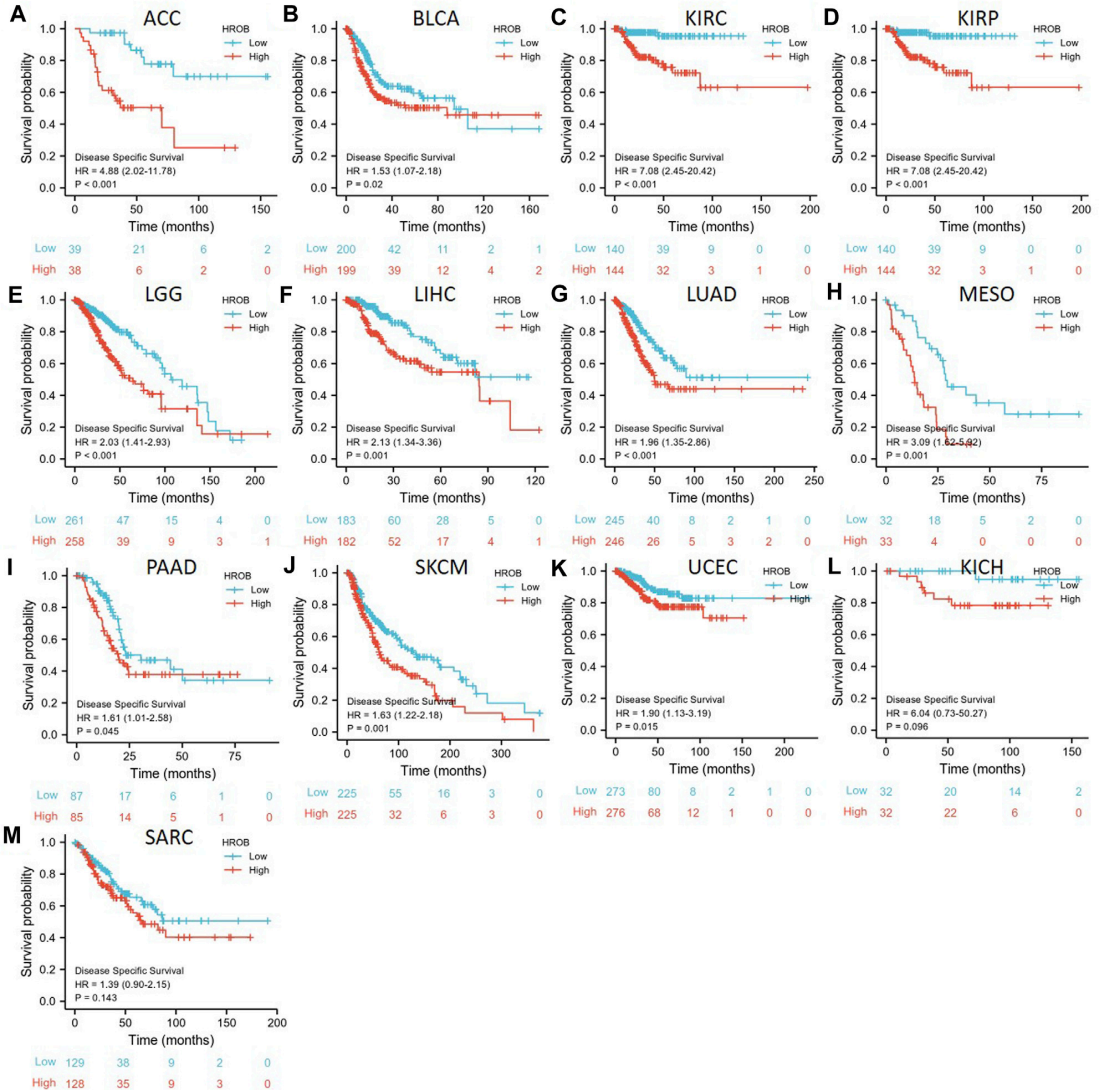
Correlation between *HROB* expression and disease-specific survival in TCGA patients with various tumor types **(A–M)**.

Given the impact of non-cancer deaths on survival analysis, we also analyzed the correlation between HROB expression and disease-specific survival (DSS) in TCGA tumor patients. Our K–M survival analysis showed that a higher expression of HROB was associated with shorter DSS in ACC (*p* < 0.001), BLCA (*p* = 0.02), KIRC (*p* = 0.002), KIRP (*p* < 0.001), LGG (*p* < 0.001), LIHC (*p* = 0.001), LUAD (*p* = 0.001), MESO (*p* < 0.001), prostate adenocarcinoma (PAAD) (*p* = 0.045), SKCM (*p* < 0.001), and UCEC (*p* = 0.022) patients (Figures 4A–K). We also found that high HROB expression might be a risk factor for KICH (*p* = 0.096) and SARC (*p* = 0.087) patients (Figures 4L–M). Raw data for the DSS analyses in Figure 4 can be found in the Supplementary Table S3. In addition, a summary table for all survival analyses can be found in Supplementary Table S2.

**FIGURE 5:**
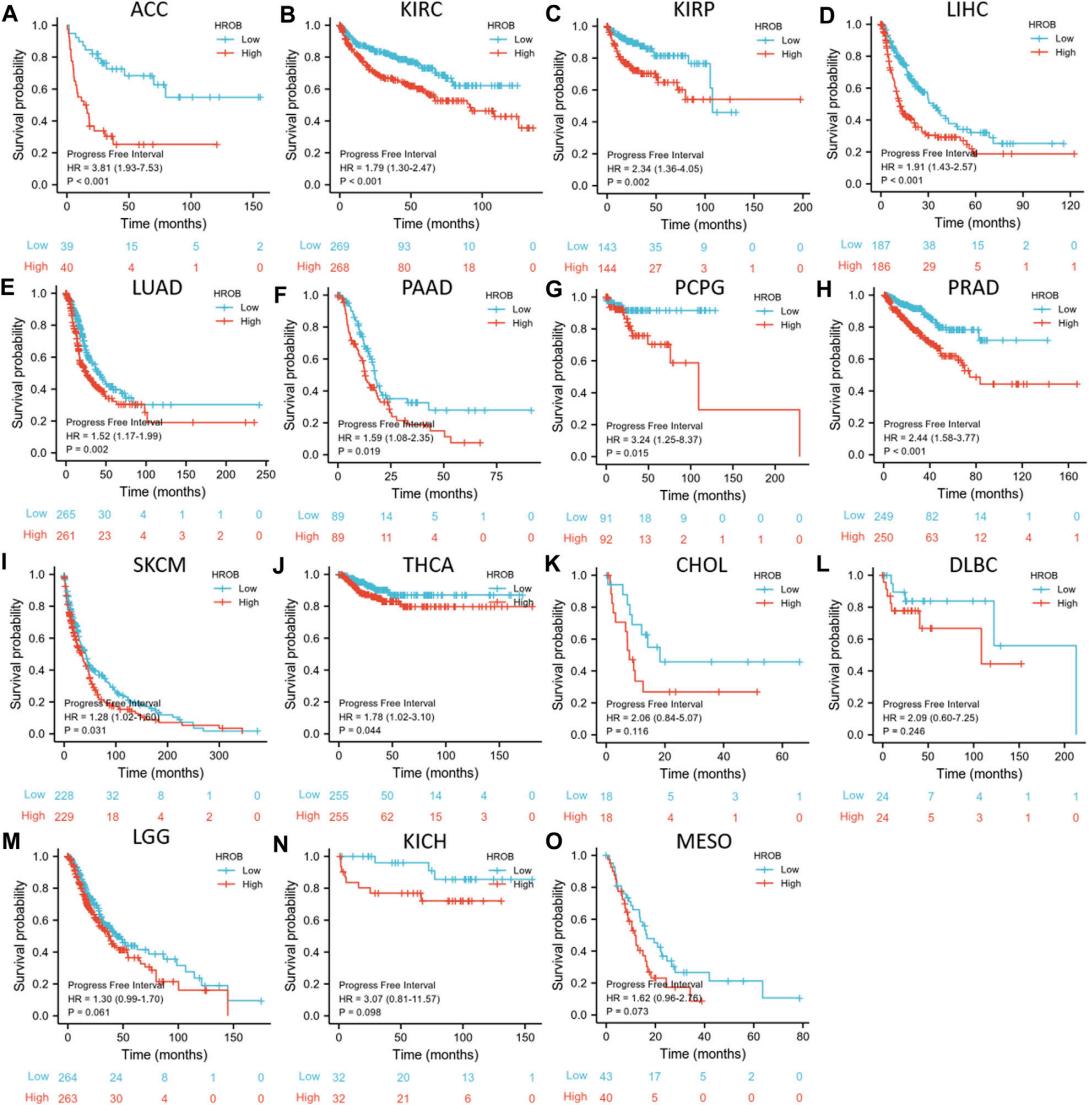
Correlation between *HROB* expression and progression-free survival in TCGA patients with various tumor types **(A–O)**.

Similar analysis was used to investigate the relationship between HROB expression and PFI in TCGA tumor patients. PFI analysis also suggested that HROB overexpression may indicate poor prognosis for tumor patients, including ACC (*p* < 0.001), KIRC (*p* < 0.001), KIRP (*p* = 0.002), LIHC (*p* < 0.001), LUAD (*p* = 0.002), PAAD (*p* = 0.019), pheochromocytoma and paraganglioma (PCPG) (*p* = 0.015), prostate adenocarcinoma (PRAD) (*p* < 0.001), SKCM (*p* = 0.031), and THCA (*p* = 0.044) (Figures 5A–J). We also found that a higher expression of HROB might be correlated with shorter PFI among patients with CHOL (*p* = 0.116), lymphoid neoplasm diffuse large B-cell lymphoma (DLBC) (*p* = 0.246), KICH (*p* = 0.098), LGG (*p* = 0.061), and MESO (*p* = 0.073) (Figures 5K–O). Raw data for the PFI analyses in Figure 5 can be found in the Supplementary Table S4. In addition, a summary table for all survival analyses can be found in Supplementary Table S2.

Collectively, our results showed that high HROB expression might be a risk prognostic factor for various tumor patients.

### HROB Expression Is Correlated With Cancer Stemness in Various Cancer Types

**FIGURE 6:**
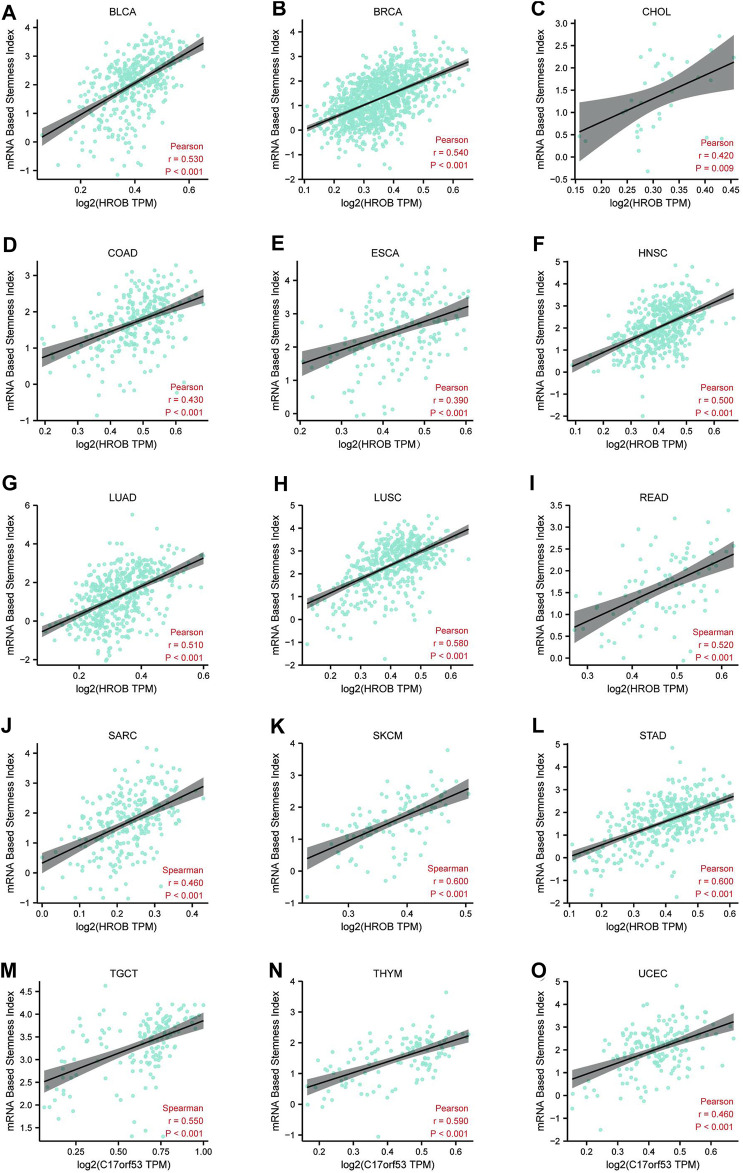
Correlation (*r* > 0.35, *p* < 0.01) between *HROB* expression and cancer stemness of various tumor types **(A–O)**.

Accumulation of dedifferentiated status and stem-cell-like characteristics are both features of tumor progression. Primary tumors with dedifferentiated phenotypes are more likely to metastasize, causing disease progression and poor outcome as metastatic tumors are often resistant to existing treatments (Gentles et al., 2010; Friedmann-Morvinski and Verma, 2014). Previously, an mRNA expression-based stemness index (mRNAsi) was developed by machine-learning algorithm to effectively measure the oncogenic dedifferentiation (Malta et al., 2018). The machine-learning algorithm was an innovative one-class logistic regression (OCLR) machine-learning algorithm, which were used to extract transcriptomic and epigenetic feature sets derived from non-transformed pluripotent stem cells and their differentiated progeny. Here, we explored the potential relationship between HROB expression and cancer stemness of TCGA tumor tissues. By applying a strict threshold (*r* > 0.35, *p* < 0.001), we identified positive correlations between HROB expression and mRNAsi of 15 tumor types including BLCA, BRCA, CHOL, COAD, ESCA, HNSC, LUAD, LUSC, READ, SARC, SKCM, STAD, testicular germ cell tumors (TGCTs), thymoma (THYM), and UCEC (Figure 6). These results implicate that HROB may promote tumorigenesis by contributing to cancer stemness in primary tumor tissues. Raw data for the above stemness analyses can be found in Supplementary Table S5. In addition, a summary table for the survival and stemness analyses can be found in Supplementary Table S2.

### Immune Infiltration Analysis of HROB

**FIGURE 7:**
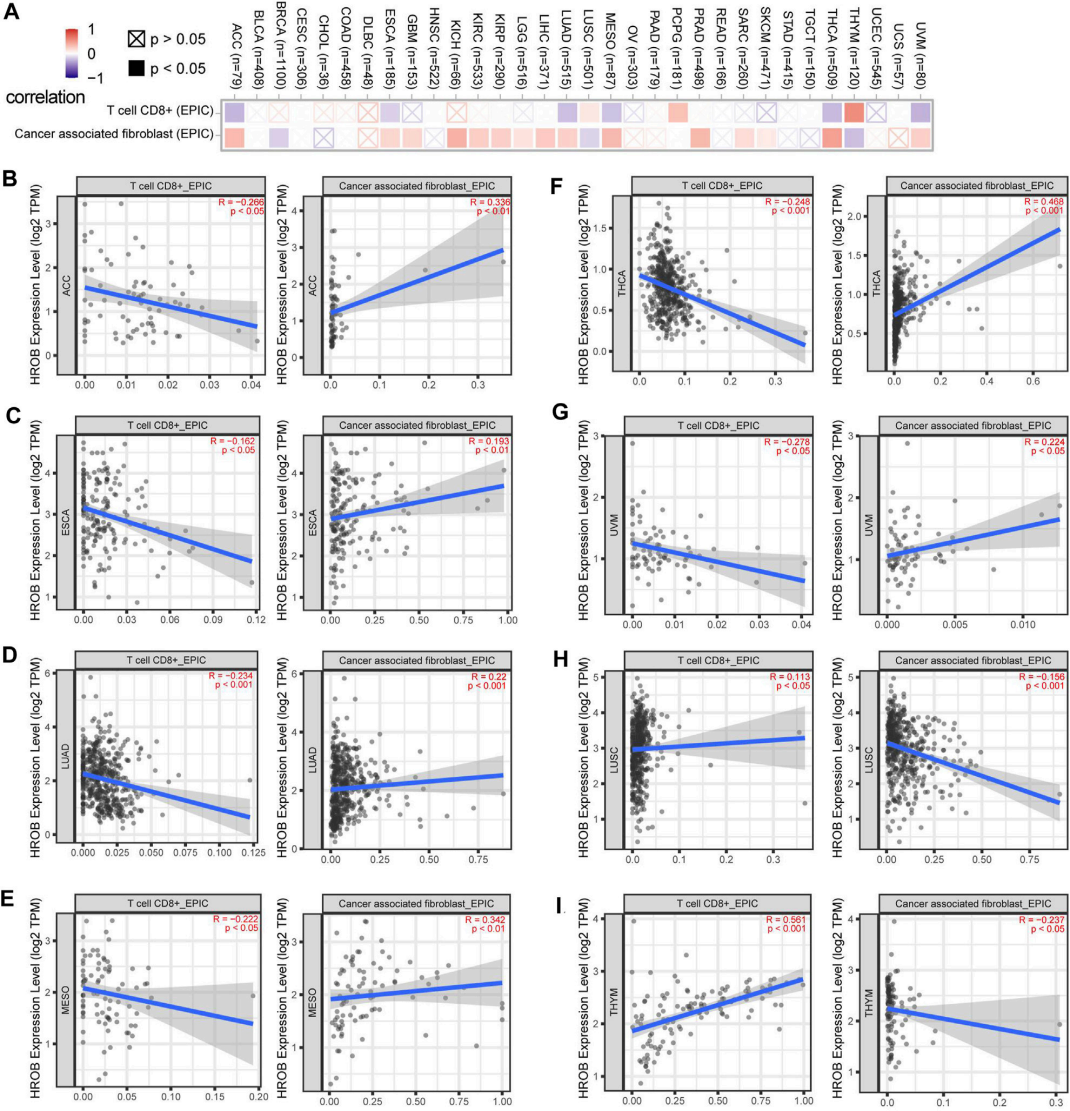
Correlation between HROB expression and cancer-associated fibroblasts, CD8+ T-cell infiltration. EPIC algorithms were used to calculate immune infiltration in all tumor types from TCGA. **(A)** Overview of the correlation between HROB expression and cancer-associated fibroblasts, CD8+ T-cell infiltration. **(B–I)** Eight tumors that are significantly correlated with both cancer-associated fibroblasts and CD8+ T-cell infiltration are displayed.

Tumor-infiltrating immune cells are key players in tumor progression, and tumors can develop strategies to intervene anti-tumor immune responses (Hiam-Galvez et al., 2021). As the most abundant stromal population, cancer-associated fibroblasts (CAFs) can suppress anti-tumor immune responses by secreting immunomodulatory factors (Erez et al., 2010; Comito et al., 2014; Harper and Sainson, 2014). Meanwhile, CD8^+^ T-cell infiltration and cytotoxicity play indispensable roles in tumor immunity (Galon et al., 2006; Gooden et al., 2011). Due to its better performance, analytical algorithm Extended Poly-dimensional Immunome Characterization (EPIC) has been recommended to explore the CAFs and CD8^+^ T-cell infiltration from bulk RNA-sequencing (RNA-seq) data (Sturm et al., 2019). In this study, we explored the potential relationship between HROB expression and infiltration status of CAFs and CD8^+^ T cell in various tumor types from TCGA by using EPIC algorithms (Racle et al., 2017). Statistically, we observed negative correlations between HROB expression level and CD8^+^ T-cell immune infiltration in ACC, ESCA, LUAD, MESO, THCA, and UVM (Figure 7A). On the contrary, significant positive correlations were observed between HROB expression and CAF infiltration for ACC, ESCA, GBM, KICH, KIRC, KIRP, LGG, LIHC, MESO, PRAD, SARC, SKCM, THCA, and UVM (Figure 7A). Recent studies have reported that CAFs could protect tumor cells by driving the deletion of CD8^+^ T cell (Lakins et al., 2018; Freeman and Mielgo, 2020). As Figures 7A–I showed, HROB expression was positively correlated with immune infiltrating levels of CAFs, and in the meantime, the HROB expression was in negative correlation with CD8^+^ T-cell infiltration (Figures 7B–G). Although it is worth noting that we observed counterexamples in LUSC and THYM (Figures 7H,I). A summary table for the above analyses can be found in Supplementary Table S2.

### HROB Is Involved in Pathways Related to Cell Cycle and Mitotic Regulation

**FIGURE 8:**
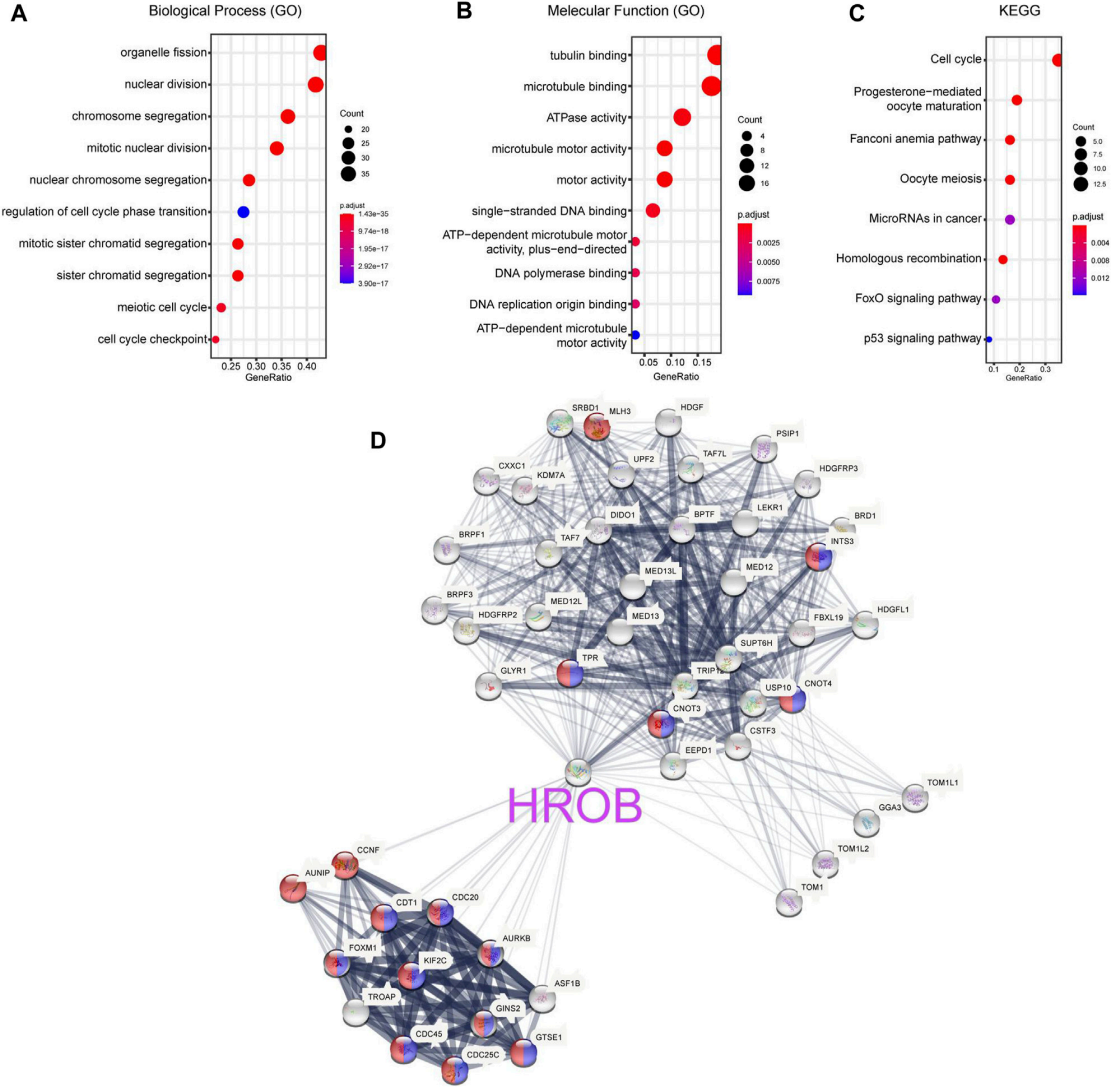
*HROB*-related gene enrichment analysis. **(A–C)** Gene Ontology (GO) and Kyoto Encyclopedia of Genes and Genome (KEGG) pathway enrichment analysis of top 100 genes co-expressed with *HROB* obtained from the GEPIA2. **(D)** Co-expression network of 50 genes co-expressed with *HROB* obtained from the STRING. The red nodes and blue nodes indicated genes in “cell cycle processes” (GO:0022402) and “mitotic cell cycle processes” (GO:1903047) of Gene Ontology, respectively.

To explore the molecular function of HROB, we used GEPIA2 to obtain 100 genes with expression pattern similar to HROB in all TCGA samples (Supplementary Table S6). Gene Ontology and Kyoto Encyclopedia of Genes and Genome (KEGG) pathway enrichment analysis suggested that these HROB-related genes were involved in cell cycle and mitotic regulation (Figures 8A–C). In addition, these results were validated when we performed the enrichment analysis of HROB co-expressed genes by using the STRING tool (Figure 8D; Supplementary Tables S7–S10). These evidences suggested that HROB is involved in DNA replication and cell proliferation, which is consistent with results of previous studies.

## Discussion

Previous studies found that HROB, previously known as uncharacterized C17ORF53, was highly conserved in vertebrates and disruption of HROB gene could induce infertility by depleting germ cells (Hustedt et al., 2019; Wang et al., 2020). HROB depletion could also inhibit the progression of DNA replication fork and cell proliferation (Wang et al., 2020). Meanwhile, HROB was involved in inter-strand crosslink repair and homologous recombination by physically interacting with the vMCM8–MCM9 helicase (Hustedt et al., 2019; Huang et al., 2020; Wang et al., 2020). Besides, several studies found that HROB may promote chemoresistance against various cancer therapeutic agents (Hustedt et al., 2019; MacLeod et al., 2019; Wang et al., 2019; Huang et al., 2020). However, although these results suggested that HROB promotes tumorigenesis in various tumor types, the evidence of these studies is relatively limited and the prevalence and predictive value of HROB in pan-cancer is unclear.

In this study, we systematically conducted pan-cancer analysis of HROB in TCGA tumor datasets. First, we examined HROB expression in tumors and corresponding non-tumor tissues and found that HROB was significantly overexpressed in up to 17 tumor types. In addition, we also found that high HROB expression was significantly correlated with the advanced stage in several tumor types. Second, our results showed that higher HROB expression may be associated with poor prognosis (OS, DSS, and PFI) in patients with various tumor types. These evidences suggested that HROB may be a potential prognostic biomarker for tumor patients. Third, our results demonstrated that the HROB expression level was significantly correlated with the cancer stemness of primary tumor tissues in TCGA datasets. The stemness index applied in the present study was derived from mRNA expression through machine-learning method, which could stratify tumor tissues by their dedifferentiation characteristics (Malta et al., 2018). Accumulation of stem-cell-like phenotype is an important characteristic, which indicate resistance to tumor therapy and poor prognosis (Gentles et al., 2010; Friedmann-Morvinski and Verma, 2014; Malta et al., 2018). Our results demonstrated that HROB may be a key molecule in promoting cancer stemness. Fourth, by applying the EPIC algorithm, we found that HROB expression is statistically associated with the infiltration level of CAFs and CD8^+^ T cells. Both of CAF and CD8^+^ T cells have been reported as key components in tumor microenvironment and have impacts on tumor immunotherapy. Finally, querying HROB co-expressed genes in GEPIA2 and STRING revealed strong correlation between HROB and a series of genes involved in cell cycle and DNA replication, such as FOXM1, CDC20, and AUNIP. The pathway-based enrichment analysis was consistent with previous studies (Hustedt et al., 2019; Huang et al., 2020; Wang et al., 2020). This study found that HROB could be a potential biomarker for multiple cancer types and may play an oncogenic role in tumorigenesis.

Although HROB has shown a potential predictive value for pan-cancer in this study, some limitations still existed. Given the evidence from the present study and previous studies, HROB is likely a gene associated with DNA replication, which is essential for cancer progression. However, the evidences of these studies were relatively implicative and the oncogenic role of HROB has not been systemically established in any tumor types. Thus, further experimental validation is still needed to characterize HROB. In addition, the clinical predictive significance of HROB has only been probed in TCGA datasets, which may induce batch effect on some of the observations in the present study and independent cohort could be used to validate our results in the future.

In summary, this study showed that HROB was overexpressed in various tumor types and associated with the advanced disease stage and poor patient prognosis. HROB also showed statistical correlation with cancer stemness and immune cell infiltration of tumor tissues. These evidences made HROB a valuable biomarker in various types, and further experimental researches are warranted.

## Data Availability

The original contributions presented in the study are included in the article/Supplementary Material. Further inquiries can be directed to the corresponding author.
